# Supplementary material for: Availability and use of institutional support programs for emergency department healthcare personnel during the COVID-19 pandemic
Source: PLoS One. 2024 Apr 16;19(4):e0298807. doi: 10.1371/journal.pone.0298807 (PMC11020772; doi:10.1371/journal.pone.0298807)
Supplement: S1 Table — (PDF) [file pone.0298807.s001.pdf]

S1 Table. Availability and Use of Institutional Support Programs in COVERED Emergency Departments, June–September 2020.

Rates of use for individual support programs include all HCP (N=1,541), while rates of use for clinical work support programs are presented for clinical HCP (n=1,158)

	Program Availability, n (%) (n = 21)	Frequency of employee use of each program, n [used] / n [available] (%)
**HCP INDIVIDUAL SUPPORT PROGRAMS**		
**Instrumental Supports**	**21 (100)**	**161/1541 (10)**
Childcare support services	16 (73)	29/1186 (2)
Elder support services	2 (10)	0/140 (0)
Transportation to and from work	9 (43)	27/642 (4)
Alternative living for self-quarantine	15 (71)	40/1057 (4)
Laundry services	13 (62)	89/934 (10)
**Emotional Supports**	**20 (95)**	**86/1474 (6)**
COVID mental health hotline	15 (71)	33/1078 (3)
Stress reduction/resilience training	17 (81)	42/1248 (3)
Social media provider support platform	9 (43)	23/630 (4)
**COVID-19 Testing for Employees**	**21 (100)**	**189/1541 (12)**
Routine asymptomatic COVID-19 testing	14 (67)	53/1007 (5)
COVID-19 testing at provider request	21 (100)	149/1541 (10)
**Work Demand Mitigation**	**21 (100)**	**202/1541 (13)**
Surge staffing plan to ED	21 (100)	123/1541 (8)
Flexible scheduling to balance demands	19 (91)	102/1390 (7)
**Financial Compensation**	**21 (100)**	**156/1541 (10)**
Financial payments for front line HCP	9 (43)	77/593 (13)
Paid time off for COVID-19 quarantine	20 (95)	91/1463 (6)
Supplemental disability for HCP affected by COVID-19	4 (19)	1/207 (1)
**ED CLINICAL WORK SUPPORT PROGRAMS**		
**COVID-19 Exposure Reduction**	**21 (100)**	**356/1158 (31)**
Telehealth for ED triage	17 (81)	101/968 (10)
Telehealth for ED patient care	21 (100)	209/1158 (18)
Self-administered swabs for patient testing	7 (33)	15/406 (4)
Team doffing of PPE for COVID-19 exposure	18 (86)	140/996 (14)
**Patient Care Services**	**21 (100)**	**574/1158 (50)**
Palliative care consultations	20 (95)	86/1110 (8)
Ethics consultations	10 (48)	10/554 (2)
24-hour social worker	21 (100)	321/1158 (28)

24-hour interpreter	21 (100)	491/1158 (42)
**Patient-Family Facilitation Due to COVID-19 Restrictions**	**21 (100)**	**319/1158 (28)**
Video-facilitated patient-family communication	19 (91)	151/1048 (14)
Audio-facilitated patient-family communication	21 (100)	274/1158 (24)
**HCP Team Communication**	**21 (100)**	**455/1158 (39)**
Team debriefing after deaths/critical incidents	20 (95)	170/1093 (16)
COVID-19 status board re: PPE and clinical procedures	20 (95)	226/1122 (20)
COVID-19 status board about volume and visits	20 (95)	303/1122 (27)
