# Supplementary material for: Availability and use of institutional support programs for emergency department healthcare personnel during the COVID-19 pandemic
Source: PLoS One. 2024 Apr 16;19(4):e0298807. doi: 10.1371/journal.pone.0298807 (PMC11020772; doi:10.1371/journal.pone.0298807)
Supplement: S2 Table — (PDF) [file pone.0298807.s002.pdf]

S2 Table. Coding Program Availability: Site Investigator Report of Program Availability and HCP Reported Program Use.

Coding program availability utilized responses from the 21 site investigators and all HCP participants (N=1,541). A program was considered available if the site investigator indicated that a program was available or if at least 2 participants indicated they had used the program.

	Number of Sites with Program Available, n (%)	Coded as Available Due to Site Investigator Reporting Availability, n (%)	Coded as Available Due to ≥2 HCP Participants Reporting Use, n (%)
**HCP INDIVIDUAL SUPPORT PROGRAMS**			
**Instrumental Supports**	**21 (100)**		
Childcare support services	16 (73)	11/16 (69)	5/16 (31)
Elder support services	2 (10)	2/2 (100)	0/2 (0)
Transportation to and from work	9 (43)	4/9 (44)	5/9 (56)
Alternative living for self-quarantine	15 (71)	12/15 (80)	3/15 (20)
Laundry services	13 (62)	5/13 (38)	8/13 (62)
**Emotional Supports**	**20 (95)**		
COVID mental health hotline	15 (71)	12/15 (80)	3/15 (20)
Stress reduction/resilience training	17 (81)	15/17 (88)	2/17 (12)
Social media provider support platform	9 (43)	6/19 (32)	13/19 (68)
**COVID-19 Testing for Employees**	**21 (100)**		
Routine asymptomatic COVID-19 testing	14 (67)	3/9 (33)	6/9 (67)
COVID-19 testing at provider request	21 (100)	4/14 (29)	10/14 (71)
**Work Demand Mitigation**	**21 (100)**		
Surge staffing plan to ED	21 (100)	16/21 (76)	5/21 (24)
Flexible scheduling to balance demands	19 (91)	9/21 (43)	12/21 (57)
**Financial Compensation**	**21 (100)**		
Financial payments for front line HCP	9 (43)	7/9 (79)	2/9 (21)
Paid time off for COVID-19 quarantine	20 (95)	19/20 (95)	1/20 (5)

Supplemental disability for HCP affected by COVID-19	4 (19)	4/4 (100)	0/4 (0)
**ED CLINICAL WORK SUPPORT PROGRAMS**			
**COVID-19 Exposure Reduction**	**21 (100)**		
Telehealth for ED triage	17 (81)	6/17 (35)	11/17 (65)
Telehealth for ED patient care	21 (100)	12/21 (57)	9/21 (43)
Self-administered swabs for patient testing	7 (33)	0/7 (0)	7/7 (100)
Team doffing of PPE for COVID-19 exposure	18 (86)	9/18 (50)	9/18 (50)
**Patient Care Services**	**21 (100)**		
Palliative care consultations	20 (95)	15/20 (75)	5/20 (25)
Ethics consultations	10 (48)	8/10 (80)	2/10 (10)
24-hour social worker	21 (100)	7/21 (33)	14/21 (67)
24-hour interpreter	21 (100)	21/21 (100)	0/21 (0)
**Patient-Family Facilitation Due to COVID-19 Restrictions**	**21 (100)**		
Video-facilitated patient-family communication	19 (91)	7/19 (37)	12/19 (63)
Audio-facilitated patient-family communication	21 (100)	13/21 (62)	8/21 (38)
**HCP Team Communication**	**21 (100)**		
Team debriefing after deaths/critical incidents	20 (95)	12/20 (60)	8/20 (40)
COVID-19 status board re: PPE and clinical procedures	20 (95)	13/20 (65)	7/20 (35)
COVID-19 status board about volume and visits	20 (95)	15/20 (75)	5/20 (25)
		Total: 267/439 (61)	Total: 172/439 (39)
