# Supplementary material for: Availability and use of institutional support programs for emergency department healthcare personnel during the COVID-19 pandemic
Source: PLoS One. 2024 Apr 16;19(4):e0298807. doi: 10.1371/journal.pone.0298807 (PMC11020772; doi:10.1371/journal.pone.0298807)
Supplement: S1 File — (PDF) [file pone.0298807.s003.pdf]

S1 File. COVID-19 Related Stress, Burnout and PTSD Risk Questionnaire

<u>**Individual Items**</u>

1. In the past week, how much has the COVID-19 pandemic affected your stress or anxiety levels?

- 1, Not at all
- 2
- 3
- 4, Somewhat
- 5
- 6
- 7, Extremely

In the past week, have you:

a) had nightmares related to the pandemic or thought about the pandemic when you did not want to? ☐ Yes ☐ No
b) tried hard not to think about the pandemic or gone out of your way to avoid situations that reminded you of it? ☐ Yes ☐ No
c) been constantly on guard, watchful, or easily startled? ☐ Yes ☐ No
d) felt numb or detached from people, activities or your surroundings? ☐ Yes ☐ No
e) felt guilty or unable to stop blaming yourself or others for the effects of the pandemic or any problems the pandemic may have caused? ☐ Yes ☐ No

****Primary Care PTSD Screen for DSM-5 adapted from: Prins, A., Bovin, M. J., Kimerling, R., Kaloupek, D. G, Marx, B. P., Pless Kaiser, A., & Schnurr, P. P. (2015). Primary Care PTSD Screen for DSM-5 (PC-PTSD-5) [Measurement instrument]. Available from https://www.ptsd.va.gov*

<u>**Program Items (completed by each participant regarding their individual use of program)**</u>

Which of the following staff-support programs have you used or received by or within your workplace during the COVID-19 pandemic? [check all that apply]

- Childcare support services
- Elder care support services
- Transportation services for employees to and from the workplace to limit public exposure
- Alternative living situation (either providing accommodations or funding for accommodations) for self-quarantine related to workplace exposure
- Laundry services for work-related clothing
- COVID-specific mental health hotline for healthcare personnel
- Stress reduction/emotional resilience training

- Routine COVID-19 testing of asymptomatic health care personnel (not including procedures for Project COVERED)
- Employee COVID-19 testing at provider request - even if asymptomatic
- Surge staffing plans for employees in other areas in the hospital to surge to the ED in response to anticipated COVID-19 patient volume [please select this item if a plan was in place and/or cross-training occurred, regardless of whether those plans were used]
- Flexible scheduling of work hours to balance demands across front-line health care personnel
- Formal use of a social media platform to facilitate communication among front-line health care personnel within your workplace (e.g., WhatsApp)
- Additional financial payments for front-line health care personnel
- Paid time off for COVID-19-related quarantine or isolation
- Supplemental disability benefits for staff affected by COVID-19

What other staff-support programs provided by or within your workplace have you used?

____

Which of the following specific clinical services have you used in your emergency department as you care for patients with COVID-19? [check all that apply]

- Telehealth for ED triage to limit provider exposure
- Telehealth for ED patient care to limit provider exposure
- Palliative care consultations
- Ethics consultations
- 24-hour social worker availability (either in-person, electronic/virtual, or on-demand)
- Self-administered swabs for patients suspected of having COVID-19 (instead of provider- or nurse collected nasopharyngeal swabs)
- Video-facilitated patient-family communication (when family are unable or prevented from being at a patient's bedside)
- Audio-facilitated patient-family communication (when family are unable or prevented from being at a patient's bedside)
- Team debriefing after ED deaths or other critical incidents
- Team doffing (e.g., each time you remove PPE related to COVID-19 exposure, a colleague observes doffing to ensure no self-contamination occurs)
- Current COVID-19 status board (in ED or on easily accessible electronic access) with updated information about PPE use and evolving clinical procedures
- Current COVID-19 status board (in ED or on easily accessible electronic access) with current COVID-19 hospitalization volume and daily visit tracking

<u>Program Items (completed by site principal investigator regarding availability of program)</u>

Which of the following programs/resources are available to support providers and staff in your workplace during the COVID-19 pandemic? [check all that apply]
If a resource was available before the COVID-19 pandemic and is still available, please indicate that it is available.

- Childcare support services
- Elder care support services

- Transportation services for employees to and from the workplace to limit public exposure
- Alternative living situation (either providing accommodations or funding for accommodations) for self-quarantine related to workplace exposure
- Laundry services for work-related clothing
- COVID-specific mental health hotline for healthcare personnel
- Stress reduction/emotional resilience training
- Routine COVID-19 testing of asymptomatic health care personnel (not including procedures for Project COVERED)
- Employee COVID-19 testing at provider request - even if asymptomatic
- Surge staffing plans for employees in other areas in the hospital to surge to the ED in response to anticipated COVID-19 patient volume [please select this item if a plan was in place and/or cross-training occurred, regardless of whether those plans were used]
- Flexible scheduling of work hours to balance demands across front-line health care personnel
- Formal use of a social media platform to facilitate communication among front-line health care personnel within your workplace (e.g., WhatsApp)
- Additional financial payments for front-line health care personnel
- Paid time off for COVID-19-related quarantine or isolation
- Supplemental disability benefits for staff affected by COVID-19

Which of the following specific clinical services are available in your emergency department as you care for patients with COVID-19? [check all that apply]
If a service was available before COVID-19 and is still available, please indicate that it is available.

- Telehealth for ED triage to limit provider exposure
- Telehealth for ED patient care to limit provider exposure
- Palliative care consultations
- Ethics consultations
- 24-hour social worker availability (either in-person, electronic/virtual, or on-demand)
- Self-administered swabs for patients suspected of having COVID-19 (instead of provider- or nurse collected nasopharyngeal swabs)
- Video-facilitated patient-family communication (when family are unable or prevented from being at a patient's bedside)
- Audio-facilitated patient-family communication (when family are unable or prevented from being at a patient's bedside)
- Team debriefing after ED deaths or other critical incidents
- Team doffing (e.g., each time you remove PPE related to COVID-19 exposure, a colleague observes doffing to ensure no self-contamination occurs)
- Current COVID-19 status board (in ED or on easily accessible electronic access) with updated information about PPE use and evolving clinical procedures
- Current COVID-19 status board (in ED or on easily accessible electronic access) with current COVID-19 hospitalization volume and daily visit tracking
