# Supplementary material for: Availability and use of institutional support programs for emergency department healthcare personnel during the COVID-19 pandemic
Source: PLoS One. 2024 Apr 16;19(4):e0298807. doi: 10.1371/journal.pone.0298807 (PMC11020772; doi:10.1371/journal.pone.0298807)
Supplement: Attachment — Submitted filename: Response to Reviews.docx [file pone.0298807.s004.docx]

**Response to Reviews**

Thank you for your consideration of our manuscript and suggestions for improvement. We appreciate the reviewer’s comments regarding the importance of the topic and the strength of high response rates from participants. We believe that the revised version is substantially improved. Below, we detail our responses to the major points raised in the review.

1. **“There is a question whether or not the limitations of the study as highlighted by the authors make the data and conclusions less generalizable even to the same population later in the pandemic.”**

We view the fact that data were collected early in the pandemic as both a potential strength and potential limitation. The timeframe is a strength in that the data are relevant to a period soon after a new significant outbreak of illness began, adding to prior work with past virus outbreaks. Prior authors have called attention to the need for healthcare staff support programing during prior outbreaks, yet the need persists. We have now added to the text of the discussion on lines 338-340 (i.e., “However, information regarding HCP needs early in the pandemic are important to prepare for the future, and the current data build on prior research from early responses during other virus outbreaks.”). Additionally, in response to the reviewer’s point #5 below, we have added to the text describing the needs of healthcare workers identified from past outbreaks. It is possible that the data may not generalize to later stages of the COVID pandemic, and we mention this as a limitation on lines 334-338: “information about institutional program use was collected only at one time point, and thus the cross-sectional nature of the data precludes an assessment of changes in program availability and use over time. It is possible that HCP with anxiety, stress, burnout, and/or PTSD symptoms used program resources later in the pandemic.”

2. **“Do the authors believe there is potential bias and lack of generalizability of the data and conclusions based on the demographics of the study volunteers and lower representation of certain groups? Do the percentages enrolled in the study reflect the percentages in health care writ large?”**

The demographic characteristics of participants in our study sample are consistent with the characteristics of hospital and outpatient clinical employees in the US in 2022, and thus we believe the data are generalizable to that population. We have added a comment about demographics of the sample and the US in general with a citation from the US Bureau of Labor Statistics. It is possible that the experience of healthcare workers in hospitals are not fully generalizable to all rural community settings, and we note this in the discussion. This text on lines 331-334 now reads: “participants were ED HCP from primarily large academic and urban centers in the US with demographic characteristics generally consistent with the US hospital workforce[42]; however, their experiences may not reflect those of community and rural ED HCP”

3. **“Are we certain there was not baseline stress and burnout at busy urban EDs prior to the pandemic? The early timepoints in the pandemic and low (relative) case load beg the question of whether the survey responses were not solely associated with the pandemic. Maybe?”**

We do believe that at baseline participants in our study likely were experiencing some stress and burnout at baseline by virtue of working in an Emergency Department. Although we do not have pre-pandemic measures in the current study, we cite prior research (lines 83-88) demonstrating that Emergency Department staff are in general at elevated risk for stress and burnout compared to many other segments of healthcare. We believe that this known risk at baseline coupled with the fact that EDs early in the pandemic faced higher risks of COVID-19 exposure makes the setting an important one to focus on regarding staff support programs.

The wording of the questions in our survey was chosen to be specific to the setting of the COVID-19 pandemic (e.g., “In the past week, how much has the COVID-19 pandemic affected your stress or anxiety levels?” “In the past week have you had nightmares related to the pandemic…”). We reasoned that this wording would reduce the likelihood of measuring baseline stress. The goal of our analyses was not to identify causes of stress or anxiety, but rather to describe the proportion of participants reporting elevated stress and anxiety at the time that we assessed support program availability. In the revised version of the manuscript, we have added text in the methods highlighting that the wording of items was specific to the pandemic (lines 155-156).

4. **Re: the abstract: “A descriptive statement on what the programs entailed would make the abstract more accessible to the general reader.”**

We agree that additional detail in the abstract would be helpful and have now added a list of the primary program types that were assessed with examples for clarification.

5. **“Literature from the west Africa ebola outbreak and how HCPs addressed issues similar to those raised in your manuscript might be worth commenting on citing. Military literature could also provide some interesting context.”**

Thank you for the suggestion to add information about prior outbreaks and from the context of the military. One study that provides important information from prior outbreaks, including Ebola, is a meta-analysis that we had previously cited in the discussion, but had failed to explain had spanned multiple outbreaks (e.g., SARS, MERS, H1N1, Ebola). This meta-analysis demonstrates the importance of integrating literature on healthcare center program responses over time from multiple outbreaks. Based on the reviewer’s suggestion, we now describe the data are from multiple prior outbreaks including listing the relevant viruses (lines 286-288). We also added text regarding the relevance of military literature, highlighting a COVID-19 healthcare worker peer-support program based on prior military interventions (lines 292-296).

That section of text now reads: “A systematic review, which included data from multiple prior virus outbreaks worldwide (i.e., severe acute respiratory syndrome [SARS], influenza caused by H1N1, Middle East respiratory syndrome [MERS], and Ebola), demonstrated that staff support protocols, clear communication, psychosocial interventions, and adequate infection protection were all important components of programming to improve the psychological wellbeing of medical staff during virus outbreaks [40]. Digital learning packages have been developed to aid in resiliency and coping in some medical centers [28]. Data regarding effective interventions to promote resilience in military settings and/or during natural disasters are relevant to inform programming during pandemics. For example, peer-support interventions developed initially for the military have also been used in the context of the COVID-19 pandemic[29], as peer-support has the advantage of being rapidly deployable and promoting interpersonal relationships. Indeed, in a qualitative study of HCP participating in the COVID-19 Pandemic and Emotional Well-Being Study, participants highlighted personal strategies for maintaining social connectedness that were important in coping with uncertainty from their work [41].”

6. **“Is there any potential that those who worked more became more comfortable with the PPE and other processes and therefore did not experience the levels of anxiety and stress those less familiar may have experienced?”**

This is an interesting alternative explanation that we had not yet considered. We have now incorporated this idea into the discussion (lines 323-325) as follows: “It is also possible that HCP who worked more hours became more familiar with use of PPE and might not have experienced the same level of concern about risk.”

7. **“Had the CARES act been implemented at this point?”**

The CARES act was signed into law on March 27, 2020, which was 3 months before the start of data collection for the current study. The scope of the CARES act was wide ranging including, for example, providing support for medical equipment manufacturers, public health programs, building medical supplies for the US Strategic Stockpile, and facilitating drug applications to the US government. Effects of the CARES act could have had an impact at the healthcare institution level, and presumably any potential trickle down to the individual would have been captured via individual responses to program use.
