# Supplementary material for: Availability and use of institutional support programs for emergency department healthcare personnel during the COVID-19 pandemic
Source: PLoS One. 2024 Apr 16;19(4):e0298807. doi: 10.1371/journal.pone.0298807 (PMC11020772; doi:10.1371/journal.pone.0298807)
Supplement: Attachment — Submitted filename: Review 2 Responses.docx [file pone.0298807.s005.docx]

1. **On line 124 it says survey participants completed the survey in "week 4 of COVID-19 surveillance between June 10 and September 23..." Please clarify if you mean you sent the survey in the 4th week of each of those months between June and September.**

Thank you for this comment. In re-reading the section describing the survey we agree that additional clarifying detail would be helpful. The survey was sent at the 4-week mark of each individual’s participation. The survey was not sent out in one batch, but rather each participant completed the items included in this analysis are *their* 4-week survey time point during that broader timeframe. We have edited the survey section to more accurately reflect this.

2. **Line 127 - 130, please clarify if participants received different surveys based on the site they worked vs a standard survey for all participants including support programs per site.**

All participants and site investigators were asked about the same list of support programs. That list was created by the study team ahead of time. Site investigators responded regarding whether their site offered each program for staff. Participants reported about whether they had personally used each program. We have added an explicit statement that all sites received the same list of programs on the survey.

The newly edited section regarding survey methods (lines 123-135) reads as follows:

“We administered an electronic survey regarding support program availability and use as part of the items that each participant completed in Week 4 of their enrollment in COVID-19 surveillance between June 10 and September 23, 2020. The list of programs included in the survey was developed *a priori* by study investigators based on a literature review and information provided to the research team from participating institutions (see **Appendix A** for survey items). All site investigators and participants were asked about the same list of programs.  Each site investigator reported whether their institution offered specific individual employee support programs and/or clinical work support programs within their ED at the time of data collection. On their week 4 survey, each study participant was asked whether they had personally used each of the programs on the list. For analysis and interpretation, program types were grouped into those targeting HCP individually and those targeting clinical workflow. These program groupings were created *a priori* by members of the study team based on the likely primary intended target of the program.”
